# Incremental diagnostic value of MRI PI-RADS classification combined with PSA density for prostate cancer

**DOI:** 10.3389/fonc.2026.1880161

**Published:** 2026-07-06

**Authors:** Xinbo Sun, Yongwei Chen, Shengtao Ling, Shide Gong, Bo Liu

**Affiliations:** Department of Urology, Taihe Hospital, Hubei University of Medicine, Shiyan, China

**Keywords:** Logistic regression, magnetic resonance imaging, PI-RADS, prostate cancer, PSAD

## Abstract

**Objective:**

To investigate the incremental diagnostic value of magnetic resonance imaging Prostate Imaging Reporting and Data System (PI-RADS) score combined with prostate-specific antigen density (PSAD) for prostate cancer (PCa), and to evaluate the clinical implications of different combined strategies.

**Methods:**

A total of 187 patients who underwent prostate MRI and had pathological results were retrospectively included, including 117 with PCa and 70 with benign prostatic hyperplasia. Univariate and multivariate logistic regression analyses were performed. A PI-RADS-only model, a log(PSAD)-only model, a combined model, and an adjusted model were constructed. Receiver operating characteristic curves and DeLong tests were used to evaluate diagnostic performance.

**Results:**

Age, log(tPSA), f/t ratio, log(PSAD), and PI-RADS score were all significantly associated with PCa diagnosis (all *P* < 0.001). After adjustment for age and f/t ratio, PI-RADS score (*OR* = 4.059, *95% CI*: 2.570–6.412, *P* < 0.001) and log(PSAD) (*OR* = 2.743, *95% CI*: 1.553–4.845, *P* < 0.001) remained independent factors. The combined model showed a higher AUC than the PI-RADS-only and log(PSAD)-only models.

**Conclusion:**

PI-RADS combined with PSAD improves diagnostic discrimination for PCa and may support different clinical strategies requiring higher specificity or sensitivity.

## 1 Introduction

Prostate cancer is one of the most common malignant tumors in men (1, 2), and its early and accurate identification is of great importance for clinical treatment decision-making and prognostic evaluation (3–5). Although serum PSA is an important indicator for prostate cancer screening and risk assessment, its specificity is limited (6–8). It is often influenced by factors such as benign prostatic hyperplasia and inflammation, which may easily lead to excessive biopsy or unnecessary clinical intervention (9–12).

With the widespread application of multiparametric MRI, the Prostate Imaging Reporting and Data System (PI-RADS) score has become an important imaging tool for prostate cancer risk assessment (13–16). Although the PI-RADS score can effectively predict the malignant risk of lesions, the use of imaging assessment alone still has certain limitations in clinical practice (17), especially in intermediate-risk lesions and gray-zone cases, where additional indicators are still needed to further optimize risk stratification.

PSAD comprehensively reflects the relationship between PSA level and prostate volume (18). Compared with PSA alone, it better represents the PSA burden relative to gland volume and is considered to have good auxiliary diagnostic value. Recent evidence has suggested that combining PSAD with PI-RADS may improve prostate cancer risk stratification and help reduce unnecessary biopsies (19). However, compared with previous studies, evidence remains limited regarding the simultaneous evaluation of model-level incremental value, adjusted diagnostic performance, and clinically interpretable fixed-threshold strategies in real-world cohorts. Based on real-world clinical data, this study aimed to evaluate the incremental value of combining the PI-RADS score with PSAD for the diagnosis of prostate cancer, and to explore the potential clinical significance of different fixed-threshold combinations. The included variables and model results are summarized in **Tables 1**–**3**.

**Table 1:** Comparison of baseline clinical and imaging characteristics between the BPH and PCa groups.

Variable	Overall (n=187)	BPH group (n=70)	PCa group (n=117)	*P value*
Age, years, mean ± SD	68.95 ± 9.04	65.74 ± 9.53	70.86 ± 8.18	<0.001
tPSA, median (IQR)	11.69 (6.45–32.20)	6.77 (4.84–12.91)	17.95 (8.89–59.05)	<0.001
fPSA, median (IQR)	1.72 (1.12–3.47)	1.57 (0.94–2.24)	1.96 (1.20–7.59)	0.002
f/t ratio, median (IQR)	0.14 (0.10–0.20)	0.18 (0.14–0.23)	0.13 (0.09–0.16)	<0.001
Prostate volume, mL, median (IQR)	49.38 (34.22–71.52)	55.88 (41.17–88.28)	43.16 (29.43–65.44)	0.004
PSAD, median (IQR)	0.25 (0.14–0.73)	0.13 (0.08–0.21)	0.41 (0.22–1.16)	<0.001
PI-RADS score, median (IQR)	4.00 (2.00–5.00)	2.00 (2.00–3.00)	5.00 (4.00–5.00)	<0.001
PI-RADS category 1, n (%)	12 (6.4)	11 (15.7)	1 (0.9)	<0.001
PI-RADS category 2, n (%)	38 (20.3)	32 (45.7)	6 (5.1)	
PI-RADS category 3, n (%)	29 (15.5)	14 (20.0)	15 (12.8)	
PI-RADS category 4, n (%)	46 (24.6)	11 (15.7)	35 (29.9)	
PI-RADS category 5, n (%)	62 (33.2)	2 (2.9)	60 (51.3)	

Continuous variables were presented as mean ± standard deviation or median (interquartile range), according to their distribution. Skewed variables were compared using the Mann–Whitney U test. Categorical variables were presented as n (%) and compared using the χ² test or Fisher’s exact test, as appropriate.

**Table 2:** Univariate logistic regression analysis of factors associated with the diagnosis of prostate cancer.

Variable	β	Standard error	Wald χ²	OR	95%CI	P value
Age	0.069	0.019	12.99	1.071	1.032–1.112	<0.001
log(tPSA)	0.867	0.179	23.371	2.38	1.675–3.383	<0.001
f/t ratio (per 0.01 increase)	-0.099	0.023	18.277	0.906	0.866–0.948	<0.001
Prostate volume	-0.008	0.004	3.564	0.992	0.983–1.000	0.059
log(PSAD)	1.242	0.218	32.548	3.463	2.260–5.305	<0.001
PI-RADS score	1.492	0.201	55.331	4.444	3.000–6.583	<0.001

β denotes the regression coefficient; Wald χ² denotes the Wald chi-square statistic; *OR* denotes the odds ratio; *CI* denotes the confidence interval; *tPSA* denotes total prostate-specific antigen; PSAD denotes prostate-specific antigen density; PI-RADS denotes the Prostate Imaging Reporting and Data System.

**Table 3:** Multivariable Logistic Regression Analysis of PI-RADS, PSAD, and the adjusted model for the diagnosis of prostate cancer.

Model	Variable	β	Standard error	Wald χ2	OR	95%CI	P value
PI-RADS-only model	PI-RADS score	1.492	0.201	55.331	4.444	3.000–6.583	<0.001
log(PSAD)-only model	log(PSAD)	1.242	0.218	32.548	3.463	2.260–5.305	<0.001
Combined model	PI-RADS score	1.404	0.226	38.612	4.072	2.615–6.342	<0.001
Combined model	log(PSAD)	1.058	0.264	16.075	2.88	1.717–4.830	<0.001
Adjusted model	Age	0.081	0.028	8.075	1.084	1.025–1.147	0.004
Adjusted model	f/t ratio (per 0.01 increase)	-0.065	0.029	4.893	0.937	0.885–0.993	0.027
Adjusted model	PI-RADS score	1.401	0.233	36.074	4.059	2.570–6.412	<0.001
Adjusted model	log(PSAD)	1.009	0.29	12.078	2.743	1.553–4.845	<0.001

β denotes the regression coefficient; Wald χ² denotes the Wald chi-square statistic; *OR* denotes the odds ratio; *CI* denotes the confidence interval; PSAD denotes prostate-specific antigen density; PI-RADS denotes the Prostate Imaging Reporting and Data System.

## 2 Methods

### 2.1 Study design and study population

This was a single-center retrospective diagnostic performance study. Patients who underwent prostate MRI at our center and ultimately obtained pathological results were consecutively included. Using pathological findings as the reference standard, patients were divided into the prostate cancer (PCa) group and the benign prostatic hyperplasia (BPH) group. A total of 273 records were initially available in the raw dataset. Among them, 86 records were excluded because of missing core clinical or imaging information required for model construction or PSAD calculation, including age, PSA-related indicators, PI-RADS score, and prostate diameters. Therefore, 187 patients were finally included in the primary analysis, comprising 117 cases of PCa and 70 cases of BPH. To evaluate the potential influence of selection bias, the excluded records were rechecked. However, the excluded records were not retained with complete baseline variables in the available cleaned analysis dataset; therefore, a reliable comparison of baseline characteristics between included and excluded patients was not feasible. The pathological endpoint of the present study was defined as overall prostate cancer, rather than clinically significant prostate cancer. Complete Gleason score or ISUP grade group information was not consistently available for all initially eligible patients; therefore, csPCa could not be reliably defined and was not analyzed as a separate endpoint.

### 2.2 Data collection and variable definitions

Data collected included age, total prostate-specific antigen (total prostate-specific antigen, tPSA), free prostate-specific antigen (free prostate-specific antigen, fPSA), free-to-total prostate-specific antigen ratio (f/t ratio), three prostate diameters, prostate volume, prostate-specific antigen density (prostate-specific antigen density, PSAD), and MRI PI-RADS score.

Prostate volume was calculated based on the anteroposterior diameter (AP), left-right diameter (LR), and craniocaudal diameter (CC) using the following formula:


Prostate  volume (mL)=0.52×AP×LR×CC/1000

The following variables were further calculated:


PSAD=tPSA/prostate volume

f/t ratio=fPSA/tPSA

Because tPSA and PSAD showed markedly skewed distributions, log(tPSA) and log(PSAD) were used for modeling in the regression and ROC analyses. To improve the clinical interpretability of the f/t ratio, it was rescaled by increments of 0.01 before being entered into the regression model.

### 2.3 Statistical analysis

Statistical analyses were performed using R software. Continuous variables were assessed for distributional characteristics. Normally distributed variables were presented as mean ± standard deviation and compared using the independent-samples t test. Skewed continuous variables were presented as median and interquartile range and compared using the Mann–Whitney U test. Categorical variables were expressed as number of cases and percentage, and comparisons between groups were performed using the χ² test or Fisher’s exact test, as appropriate. The pathological outcome of overall prostate cancer was used as the dependent variable (PCa = 1, BPH = 0). Because complete Gleason score or ISUP grade group information was not available for all eligible patients, no separate model for clinically significant prostate cancer was constructed. Univariate logistic regression analysis was first performed to calculate the regression coefficient (β), standard error (SE), Wald χ², odds ratio (OR), and 95% CI. In accordance with the study objective and clinical relevance, a PI-RADS-only model, a log(PSAD)-only model, a PI-RADS + log(PSAD) combined model, and an adjusted model including age, f/t ratio, PI-RADS score, and log(PSAD) were established. Considering the strong correlations among tPSA, fPSA, f/t ratio, prostate volume, and PSAD, these variables were not entered simultaneously into the same multivariable model in order to avoid multicollinearity; variance inflation factors (variance inflation factor, VIF) were used to evaluate collinearity.

Receiver operating characteristic (ROC) curves were used to assess the diagnostic performance of different models, and the area under the curve (AUC) with 95% CI was calculated. Differences in AUC between ROC curves were compared using the DeLong test. The optimal cutoff value for the predicted probability of each model was determined based on the Youden index, and the corresponding sensitivity and specificity were calculated. The optimal cutoff values and corresponding diagnostic performance are summarized in **Table 4**. In addition, fixed-threshold analysis was performed using the commonly applied clinical thresholds of PI-RADS ≥ 4 and PSAD ≥ 0.15 ng/mL/mL, with sensitivity, specificity, positive predictive value (positive predictive value, PPV), negative predictive value (negative predictive value, NPV), accuracy, and Youden index being calculated accordingly. A two-sided P < 0.05 was considered statistically significant.

**Table 4:** Optimal cutoff values and corresponding diagnostic performance of different ROC models.

Model	Optimal cutoff value	Sensitivity	Specificity
PI-RADS	0.644	0.812	0.814
log(PSAD)	0.627	0.692	0.857
PI-RADS + log(PSAD)	0.677	0.855	0.871

The optimal cutoff value was determined based on the Youden index; the cutoff values shown in the table represent the predicted probability thresholds of the models.

## 3 Results

### 3.1 Baseline characteristics

A total of 187 patients were included in this study, including 70 patients with BPH and 117 patients with PCa. Among the 273 initially available records, 86 were excluded because of missing core clinical or imaging information required for model construction or PSAD calculation. After rechecking the available cleaned analysis dataset, we found that the excluded records were not retained with complete baseline variables. Therefore, a formal comparison of baseline characteristics between included and excluded patients could not be reliably performed. The possibility of selection bias due to missing data was acknowledged in the interpretation of the results. Group comparisons showed that patients in the PCa group were older and had higher serum tPSA, fPSA, PSAD, and PI-RADS scores, whereas the f/t ratio was lower. Because tPSA, fPSA, f/t ratio, prostate volume, PSAD, and PI-RADS score showed skewed distributions, median and interquartile range values were reported and Mann–Whitney U tests were used for group comparisons. The median PSAD was significantly higher in the PCa group than in the BPH group [0.41 (0.22–1.16) vs. 0.13 (0.08–0.21), *P* < 0.001]. In terms of imaging findings, the distribution of PI-RADS categories also differed significantly between the two groups (*P* < 0.001). Specifically, in the PCa group, the proportions of patients with PI-RADS categories 4 and 5 were relatively high, accounting for 29.9% and 51.3%, respectively; in contrast, patients in the BPH group were mainly distributed in PI-RADS categories 1 and 2, accounting for 15.7% and 45.7%, respectively. These findings suggest that age, PSA-related indicators, prostate volume, PSAD, and PI-RADS score differed between the PCa and BPH groups.

### 3.2 Univariate logistic regression

Univariate logistic regression analysis showed that age, log(*tPSA*), *f/t* ratio, log(PSAD), and PI-RADS score were all significantly associated with the diagnosis of prostate cancer (all *P* < 0.001). Specifically, for each 1-year increase in age, the risk of prostate cancer increased by 7.1% (*OR* = 1.071, *95% CI*: 1.032–1.112); for each 1-unit increase in log(*tPSA*), the risk of prostate cancer increased by 2.380-fold (*OR* = 2.380, *95% CI*: 1.675–3.383); and for each 1-unit increase in log(PSAD), the risk of prostate cancer increased by 3.463-fold (*OR* = 3.463, *95% CI*: 2.260–5.305). PI-RADS score had the largest regression coefficient and Wald χ² value (β = 1.492, Wald χ² = 55.331), indicating that it had the strongest univariate predictive value for the diagnosis of prostate cancer. The *f/t* ratio was a protective factor; for each 0.01 increase, the risk of prostate cancer decreased by 9.4% (*OR* = 0.906, *95% CI*: 0.866–0.948, *P* < 0.001). The association between prostate volume and the diagnosis of prostate cancer did not reach statistical significance (*P* = 0.059).

### 3.3 Multivariable logistic regression

Multivariable logistic regression analysis showed that both the PI-RADS-only model and the log(PSAD)-only model could independently predict prostate cancer. The *OR* of the PI-RADS-only model was 4.444 (*95% CI*: 3.000–6.583, *P* < 0.001), and the *OR* of the log(PSAD)-only model was 3.463 (*95% CI*: 2.260–5.305, *P* < 0.001). In the combined model, both PI-RADS score and log(PSAD) remained statistically significant, with *OR*s of 4.072 (*95% CI*: 2.615–6.342, *P* < 0.001) and 2.880 (*95% CI*: 1.717–4.830, *P* < 0.001), respectively, indicating that the combination of the two could jointly improve the diagnostic performance for prostate cancer.

After further adjustment for age and *f/t* ratio, age, *f/t* ratio, PI-RADS score, and log(PSAD) all remained independent factors associated with the diagnosis of prostate cancer. Specifically, for each 1-year increase in age, the risk of prostate cancer increased by 8.4% (*OR* = 1.084, *95% CI*: 1.025–1.147, *P* = 0.004); for each 0.01 increase in the *f/t* ratio, the risk of prostate cancer decreased by 6.3% (*OR* = 0.937, *95% CI*: 0.885–0.993, *P* = 0.027); for each 1-point increase in PI-RADS score, the risk of prostate cancer increased by 4.059-fold (*95% CI*: 2.570–6.412, *P* < 0.001); and for each 1-unit increase in log(PSAD), the risk of prostate cancer increased by 2.743-fold (*95% CI*: 1.553–4.845, *P* < 0.001). Collinearity diagnostics showed that the variance inflation factors (VIFs) for age, *f/t* ratio, PI-RADS score, and log(PSAD) were 1.100, 1.084, 1.073, and 1.104, respectively, all close to 1, indicating no obvious multicollinearity among these variables. Therefore, they could be included in the same multivariable logistic regression model for analysis (**Supplementary Table 1**).

### 3.4 ROC curves

**Figure 1:**
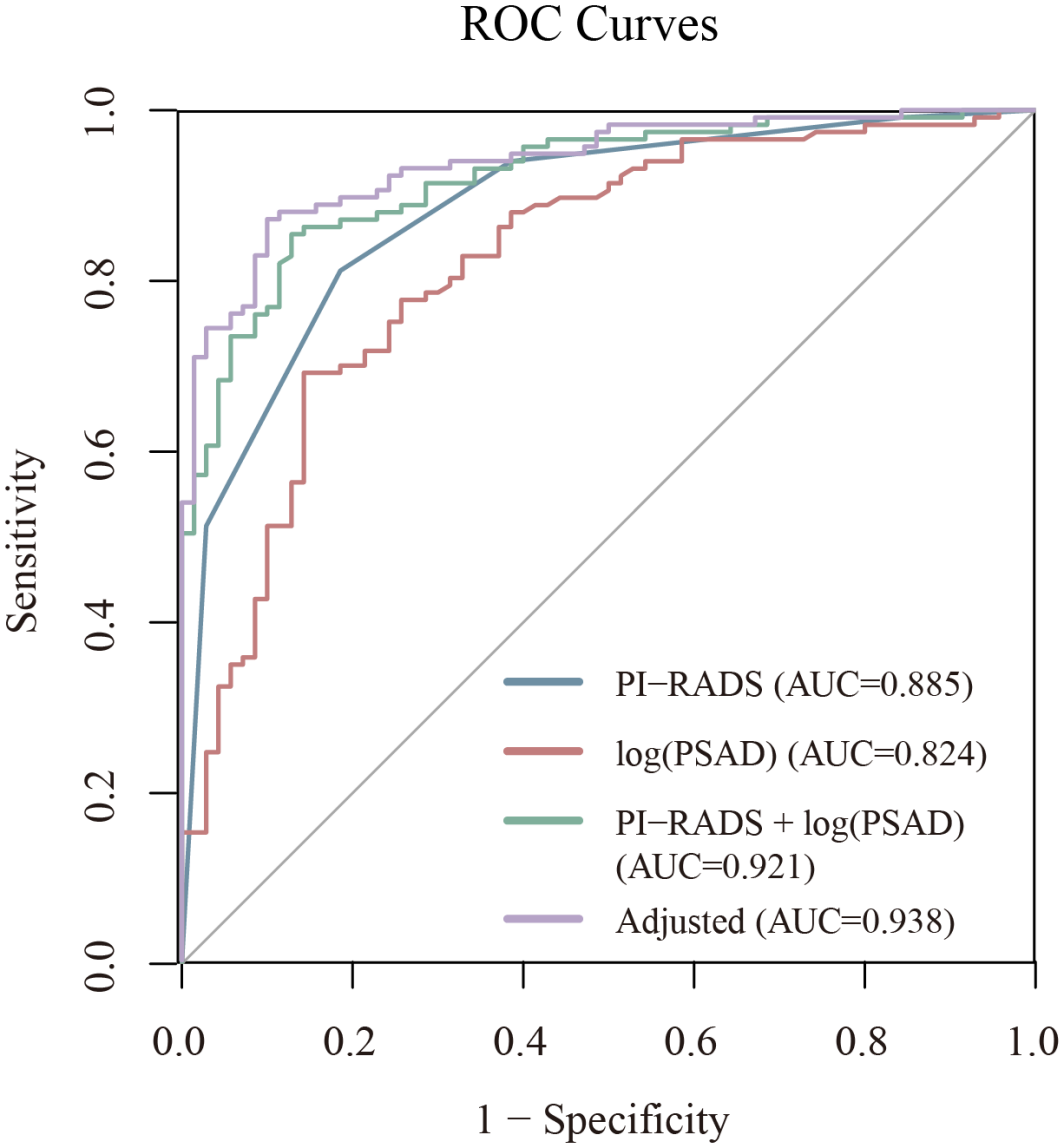
ROC curves of different models for the diagnosis of prostate cancer.

ROC curve analysis showed that PI-RADS, log(PSAD), PI-RADS combined with log(PSAD), and the adjusted model all had good diagnostic performance for prostate cancer. The AUC of the PI-RADS-only model was 0.885 (*95% CI*: 0.837–0.932), while the AUC of the log(PSAD)-only model was 0.824 (*95% CI*: 0.761–0.886). When PI-RADS and log(PSAD) were combined in the model, the AUC increased to 0.921 (*95% CI*: 0.883–0.958), indicating that the combined model had better discriminative ability than either single indicator alone. After further adjustment for age and *f/t* ratio, the AUC of the adjusted model further increased to 0.938 (*95% CI*: 0.905–0.970), suggesting that this model had the best diagnostic performance (**Table 5**, **Figure 1**).

**Table 5:** ROC analysis of different models for the diagnosis of prostate cancer.

Model	AUC	95%CI
PI-RADS	0.885	0.837–0.932
log(PSAD)	0.824	0.761–0.886
PI-RADS + log(PSAD)	0.921	0.883–0.958
Adjusted model	0.938	0.905–0.970

AUC denotes the area under the receiver operating characteristic curve; PSAD denotes prostate-specific antigen density; the adjusted model included age, *f/t* ratio, PI-RADS score, and log(PSAD).

### 3.5 Comparison of ROC curves among different models

The DeLong test showed that the difference in AUC between the PI-RADS-only model and the log(PSAD)-only model was not statistically significant (*Z* = 1.523, *P* = 0.128). Compared with the PI-RADS-only model, the AUC of the PI-RADS + log(PSAD) model was significantly higher (*Z* = −2.317, *P* = 0.021); compared with the log(PSAD)-only model, the combined model also showed a significantly larger AUC (*Z* = −3.498, *P* < 0.001). After further incorporating age and the *f/t* ratio to construct the adjusted model, its AUC was further improved compared with that of the PI-RADS-only model (*Z* = −2.908, *P* = 0.004), the log(PSAD)-only model (*Z* = −3.980, *P* < 0.001), and the combined model (*Z* = −2.003, *P* = 0.045). These results indicate that combining PI-RADS with PSAD can significantly improve the diagnostic performance for prostate cancer, and that adding age and the *f/t* ratio can further enhance the discriminative ability of the model. The pairwise ROC comparisons are summarized in **Table 6**.

**Table 6:** DeLong test for comparing the ROC curves of different models.

Comparison	Z value	P value
PI-RADS vs log(PSAD)	1.523	0.128
PI-RADS vs PI-RADS + log(PSAD)	-2.317	0.021
PI-RADS vs Adjusted	-2.908	0.004
log(PSAD) vs PI-RADS + log(PSAD)	-3.498	<0.001
log(PSAD) vs Adjusted	-3.980	<0.001
PI-RADS + log(PSAD) vs Adjusted	-2.003	0.045

The DeLong test was used to compare the differences in the areas under two correlated ROC curves.

The optimal cutoff value for each model was determined based on the Youden index. The results showed that the optimal cutoff value for the PI-RADS-only model was 0.644, at which the sensitivity was 81.2% and the specificity was 81.4%; the optimal cutoff value for the log(PSAD)-only model was 0.627, at which the sensitivity was 69.2% and the specificity was 85.7%; and the optimal cutoff value for the PI-RADS + log(PSAD) model was 0.677, at which the sensitivity was 85.5% and the specificity was 87.1%. Compared with the single-indicator models, the combined model achieved a better balance between sensitivity and specificity, further suggesting that PI-RADS combined with PSAD has better diagnostic performance.

### 3.6 Threshold analysis

Diagnostic performance was analyzed using PI-RADS ≥ 4 and PSAD ≥ 0.15 as fixed clinical thresholds. The fixed-threshold diagnostic performance results are summarized in **Table 7**. The results showed that PI-RADS ≥ 4 had a sensitivity of 81.2% and a specificity of 81.4%, indicating a good balance between the two. PSAD ≥ 0.15 had a sensitivity of 88.0%, which was higher than that of PI-RADS ≥ 4, but its specificity decreased to 61.4%. When a serial strategy (both criteria met) was applied, specificity increased to 95.7%, the positive predictive value increased to 96.7%, accuracy was 82.4%, and the Youden index was the highest (70.1%). In contrast, when a parallel strategy (either criterion met) was used, sensitivity further increased to 94.9%, and the negative predictive value was 84.6%, but specificity decreased to 47.1%. These findings suggest that the combined application of PI-RADS and PSAD can further optimize prostate cancer risk stratification: the serial strategy is more suitable for improving specificity and potentially reducing unnecessary biopsies in selected patients, although this requires further validation using csPCa as the endpoint, whereas the parallel strategy is more suitable for improving sensitivity and reducing missed diagnoses.

**Table 7:** Diagnostic performance of different combinations of PI-RADS ≥ 4 and PSAD ≥ 0.15 for prostate cancer.

Rule	Sensitivity (%)	Specificity (%)	PPV (%)	NPV (%)	Accuracy (%)	Youden index (%)
PI-RADS ≥4	81.2	81.4	88	72.2	81.3	62.6
PSAD ≥0.15	88	61.4	79.2	75.4	78.1	49.5
Both criteria met	74.4	95.7	96.7	69.1	82.4	70.1
Either criterion met	94.9	47.1	75	84.6	77	42

## 4 Discussion

Based on real-world clinical data, this study systematically evaluated the incremental diagnostic value of combining the PI-RADS score with PSAD for prostate cancer. The main findings are as follows. First, patients in the PCa group were older and had higher tPSA, fPSA, PSAD, and PI-RADS scores, whereas the f/t ratio was lower, suggesting that these clinical, laboratory, and imaging indicators were closely associated with the diagnosis of prostate cancer. Because tPSA and PSAD showed markedly skewed distributions, median and interquartile range values were used to better describe their baseline distributions, and log-transformed variables were used in regression and ROC analyses to reduce the influence of extreme values. Second, both univariate and multivariable logistic regression analyses showed that PI-RADS score and log(PSAD) were independent factors associated with the diagnosis of PCa, and both remained statistically significant after adjustment for age and *f/t* ratio. Third, ROC analysis showed that the AUC was 0.885 for the PI-RADS-only model and 0.824 for the log(PSAD)-only model, whereas the AUC increased to 0.921 for the PI-RADS + log(PSAD) model. The DeLong test further confirmed that the diagnostic performance of the combined model was significantly better than that of either the PI-RADS-only model or the log(PSAD)-only model. Finally, in the fixed clinical threshold analysis, different combined strategies showed distinct diagnostic characteristics: when both PI-RADS ≥ 4 and PSAD ≥ 0.15 were satisfied, specificity and positive predictive value were the highest; when either criterion was satisfied, sensitivity was the highest. These findings indicate that the combination of the two provides not only a statistical gain, but also clear clinical utility.

Previous studies have generally recognized that the PI-RADS score is an important tool for imaging-based risk assessment of prostate cancer (20–22), whereas PSAD can serve as an important complementary indicator beyond imaging, particularly in biopsy decision-making and the evaluation of gray-zone cases (23–25). The findings of the present study are consistent with this overall understanding. First, the PI-RADS-only model already demonstrated high diagnostic performance, indicating that the standardized MRI scoring system remains a core tool for identifying prostate cancer. Second, the log(PSAD)-only model also showed good discriminative ability, suggesting that PSAD can, to some extent, compensate for the limited specificity of PSA alone. More importantly, the present study showed that the AUC further increased after combining PI-RADS with PSAD, and the DeLong test confirmed that the combined model had a statistically significant advantage over either single model. This is in line with the previous research trend showing that a combination of imaging and clinical indicators performs better than any single indicator alone.

Several recent studies have investigated the combined use of PI-RADS and PSAD for prostate cancer risk stratification and biopsy decision-making (19, 23, 24). Consistent with these studies, the present study confirmed that combining PI-RADS with PSAD improved diagnostic discrimination compared with either indicator alone. However, the novelty of the present study lies in its simultaneous evaluation of several complementary diagnostic aspects. First, we directly compared the PI-RADS-only, log(PSAD)-only, PI-RADS + log(PSAD), and adjusted models using ROC analysis and DeLong testing. Second, log-transformed PSAD was used in the regression and ROC models to reduce the influence of skewed distribution and extreme values. Third, beyond model-level AUC improvement, this study further translated the combined assessment into clinically interpretable fixed-threshold strategies based on PI-RADS ≥ 4 and PSAD ≥ 0.15. When both criteria were satisfied, specificity reached 95.7% and PPV reached 96.7%; when either criterion was satisfied, sensitivity reached 94.9%. Therefore, the contribution of the present study is not simply to confirm the usefulness of PI-RADS combined with PSAD, but to demonstrate both its statistical incremental value and its potential decision-rule implications in a real-world clinical cohort.

The complementary nature of PI-RADS and PSAD may be related to the fact that they reflect different dimensions of prostate cancer risk. PI-RADS scoring is mainly based on MRI features and integrates information on lesion morphology, diffusion restriction, and dynamic contrast enhancement; in essence, it reflects the imaging manifestations of malignancy. In contrast, PSAD combines the serum PSA level with prostate volume and reflects the PSA burden per unit volume of prostate tissue, which is more closely related to the relationship between tumor biological activity and the background gland volume. Therefore, PI-RADS and PSAD do not provide completely overlapping information; rather, they characterize tumor risk from the perspectives of imaging and laboratory indicators, respectively. This may explain why both remained independently significant after combination and why their joint use provided incremental value.

In addition, PSAD showed a markedly right-skewed distribution and was easily influenced by extreme values when presented only as mean ± standard deviation. Therefore, median and interquartile range values were reported in the baseline table, and log(PSAD) was used in regression and ROC analyses. This approach allowed the diagnostic value of PSAD to be evaluated more robustly and reduced the influence of extreme values on model estimation.

Age and the *f/t* ratio also remained statistically significant in the adjusted model, which is consistent with the general pattern of prostate cancer risk assessment. Increasing age usually indicates a higher risk of disease, whereas a lower *f/t* ratio often suggests a greater likelihood of malignancy. However, in light of the focus of this study, these variables are more appropriately regarded as supplementary adjustment factors rather than replacing the central role of PI-RADS and PSAD in the main text.

The findings of this study suggest that the combination of PI-RADS and PSAD has clear potential for clinical application. For patients with a higher PI-RADS score, a concomitant increase in PSAD indicates a further increase in malignant risk and may strengthen clinicians’ confidence in identifying high-risk lesions. For patients in the PI-RADS gray zone, especially those with a PI-RADS score of 3, PSAD may provide additional stratification information and thus help reduce unnecessary biopsies or delayed diagnosis. For patients in the PI-RADS gray zone, especially those with a PI-RADS score of 3, PSAD may theoretically provide additional stratification information; however, this issue still requires further validation in studies with larger gray-zone samples.

The fixed-threshold analysis further demonstrates the clinical applicability of this combined strategy. When both PI-RADS ≥ 4 and PSAD ≥ 0.15 are satisfied, specificity and PPV are the highest, making this strategy more suitable for identifying patients with a higher probability of pathological PCa and reducing unnecessary biopsies. In contrast, when either criterion is met, sensitivity is the highest, making this strategy more suitable for clinical settings in which minimizing missed diagnoses is prioritized. In other words, the combination of PI-RADS and PSAD can not only improve overall diagnostic performance, but also allow flexible adjustment of the application strategy according to different clinical objectives.

However, because the present findings were derived from a single-center cohort, these threshold-based strategies should be regarded as exploratory and center-specific. Their clinical applicability requires further confirmation in external cohorts before being adopted as general biopsy decision rules.

This study has several limitations. First, as a single-center retrospective study, it is subject to a certain degree of selection bias, and the representativeness of the sample is limited. Second, 86 of the 273 initially available records were excluded because of missing core clinical or imaging information required for model construction or PSAD calculation. Although we rechecked the available cleaned analysis dataset, the excluded records were not retained with complete baseline variables, and a reliable comparison between included and excluded patients could not be performed. Therefore, the possibility of selection bias due to missing data cannot be fully excluded, and the findings should be interpreted cautiously. Third, the study outcome was defined as overall PCa versus BPH rather than clinically significant prostate cancer. Although csPCa is more relevant for contemporary biopsy decision-making and treatment-oriented risk stratification, complete Gleason score or ISUP grade group information was not consistently available for all initially eligible patients in this retrospective dataset. Therefore, csPCa could not be reliably defined or analyzed as a separate endpoint. This limitation affects the clinical utility of the proposed models: the findings should be interpreted as supporting overall PCa detection and preliminary biopsy decision-making, rather than as evidence that the models can accurately identify csPCa. Further studies incorporating complete Gleason score or ISUP grade group data are required to validate the value of PI-RADS combined with PSAD for csPCa detection. Fourth, the sample size of patients with PI-RADS 3 lesions was relatively small; therefore, the potential value of PSAD for further stratifying MRI gray-zone cases could not be fully evaluated and requires validation in larger samples. Fifth, this study was conducted at a single center and no external validation was performed. The diagnostic performance observed in this cohort may be influenced by center-specific factors, including MRI acquisition protocols, image quality, radiologist experience, PI-RADS interpretation, biopsy indications, biopsy techniques, and pathological sampling procedures. Because these factors may vary substantially across institutions, the AUC values and fixed-threshold performance reported in the present study should not be directly generalized to other clinical settings without further validation. Future studies should validate the PI-RADS + PSAD strategy in independent and preferably multicenter cohorts with standardized MRI interpretation and biopsy protocols.

In summary, the PI-RADS score has high value in the diagnosis of prostate cancer, and PSAD can provide significant incremental diagnostic information on this basis. The combination of PI-RADS and PSAD not only improves the discriminative ability of the model, but can also meet different clinical needs for either improving specificity or improving sensitivity through different threshold combinations. Future studies are still needed to further validate the stability of this combined strategy in larger samples, multicenter settings, and external validation cohorts. In addition, after incorporating Gleason score/ISUP grading information, its value for identifying clinically significant prostate cancer should be further evaluated.

## Data Availability

The raw data supporting the conclusions of this article will be made available by the authors, without undue reservation.
