# Supplementary material for: Incremental diagnostic value of MRI PI-RADS classification combined with PSA density for prostate cancer
Source: Front Oncol. 2026 Jul 6;16:1880161. doi: 10.3389/fonc.2026.1880161 (PMC13381260; doi:10.3389/fonc.2026.1880161)
Supplement: Supplementary file 1 [file Table1.docx]

**Supplementary Material**

**Table S1. Collinearity Diagnostics for the Multivariable Model**

| Variable | **VIF** |
| --- | --- |
| Age | 1.1 |
| f/t ratio (per 0.01 increase) | 1.084 |
| PI-RADS score | 1.073 |
| log(PSAD) | 1.104 |

Note: VIF denotes the variance inflation factor. All VIF values were close to 1, indicating no obvious multicollinearity among the variables.
